# Automated collection of imaging and phenotypic data to centralized and distributed data repositories

**DOI:** 10.3389/fninf.2014.00060

**Published:** 2014-06-05

**Authors:** Margaret D. King, Dylan Wood, Brittny Miller, Ross Kelly, Drew Landis, William Courtney, Runtang Wang, Jessica A. Turner, Vince D. Calhoun

**Affiliations:** ^1^The Mind Research NetworkAlbuquerque, NM, USA; ^2^Department of Psychology, Georgia State UniversityAtlanta, GA, USA; ^3^Departments of Electrical and Computer Engineering, Neurosciences, Computer Science, and Psychiatry, University of New MexicoAlbuquerque, NM, USA

**Keywords:** assessment data collection, neuroinformatics, tool suite, database, intuitive, COINS

## Abstract

Accurate data collection at the ground level is vital to the integrity of neuroimaging research. Similarly important is the ability to connect and curate data in order to make it meaningful and sharable with other investigators. Collecting data, especially with several different modalities, can be time consuming and expensive. These issues have driven the development of automated collection of neuroimaging and clinical assessment data within COINS (Collaborative Informatics and Neuroimaging Suite). COINS is an end-to-end data management system. It provides a comprehensive platform for data collection, management, secure storage, and flexible data retrieval (Bockholt et al., 2010; Scott et al., 2011). It was initially developed for the investigators at the Mind Research Network (MRN), but is now available to neuroimaging institutions worldwide. Self Assessment (SA) is an application embedded in the Assessment Manager (ASMT) tool in COINS. It is an innovative tool that allows participants to fill out assessments via the web-based Participant Portal. It eliminates the need for paper collection and data entry by allowing participants to submit their assessments directly to COINS. Instruments (surveys) are created through ASMT and include many unique question types and associated SA features that can be implemented to help the flow of assessment administration. SA provides an instrument queuing system with an easy-to-use drag and drop interface for research staff to set up participants' queues. After a queue has been created for the participant, they can access the Participant Portal via the internet to fill out their assessments. This allows them the flexibility to participate from home, a library, on site, etc. The collected data is stored in a PostgresSQL database at MRN. This data is only accessible by users that have explicit permission to access the data through their COINS user accounts and access to MRN network. This allows for high volume data collection and with minimal user access to PHI (protected health information). An added benefit to using COINS is the ability to collect, store and share imaging data *and* assessment data with no interaction with outside tools or programs. All study data collected (imaging and assessment) is stored and exported with a participant's unique subject identifier so there is no need to keep extra spreadsheets or databases to link and keep track of the data. Data is easily exported from COINS via the Query Builder and study portal tools, which allow fine grained selection of data to be exported into comma separated value file format for easy import into statistical programs. There is a great need for data collection tools that limit human intervention and error while at the same time providing users with intuitive design. COINS aims to be a leader in database solutions for research studies collecting data from several different modalities.

## Introduction

Collecting phenotypic data is a central part of any neuroimaging study. Traditionally, this data has been collected by writing observations and responses on paper. In some cases, study staff will record the data on paper while interviewing the participant. In other cases, the participant may enter the data directly onto the paper themselves. After this initial data collection, the paper hard-copies must be carefully cataloged and stored in filing systems. Since data contained on sheets of paper is difficult to analyze, the data must then be entered into a computer system (e.g., database or spreadsheet). In order to reduce errors, many studies will perform dual entry, where the data is redundantly entered by two individuals. The two entries are then compared, and any differences are resolved before an official entry is created. Even with dual entry, there is a small chance of data entry errors.

Fortunately, modern technology has provided researchers with many alternatives to the expensive, time-consuming process described above. Data collection services like SurveyMonkey^1^, Mechanical Turk, and Qualtrics^2^ (Buhrmester et al., 2011) offer comprehensive form building tools. Once built, a form can be used by study staff or a participant to enter data directly into a computer, thus avoiding the cost and time associated with entering data on paper records into a computer system. The data collected in using these systems must still be securely tied to each study participant, and their imaging data (typically stored on local databases, with metadata contained in spreadsheets). Managing the connections between electronic phenotypic data and the participant records in a way that does not compromise participant privacy is a stressful and time consuming task.

In this article we introduce the web-based Self Assessment tool as an optimal method for assessment data collection. The impetus for developing this tool was to reduce data collection and entry time as well as reduce the probability of entry errors and data loss. Accurate data collection and entry is necessary to the success of any research study. Similarly important is collection of item-level data rather than summary values. This allows researchers greater opportunity for discovery within a larger, more robust dataset (Nooner et al., 2012). Self Assessment enables researchers to collect and store all item-level assessment data in an efficient and timely way.

There are many facets to this tool that produce an easy-to-use interface and efficient data collection. Ease of use is one of the most important aspects considered while creating this tool - to reduce the time, energy, frustration of participants. The Self Assessment tool (SA) provides research staff an assessment queueing system, the ability to create user friendly instruments, the ability to review participant submitted assessments and easily export options. With this tool and others, COINS is striving to create an efficient, comprehensive and intuitive database to offer the research community.

## Methods

### Coins overview

COINS, created and developed at the Mind Research Network (MRN; The Mind Research Network for Neurodiagnostic Discovery, 2013), is a web-based data management system. COINS is unique in that it offers tools to collect, manage and share data of different modalities, including MRI, MEG, EEG and assessment data (Bockholt et al., 2010; Scott et al., 2011). There are similar neuroimaging suites (Marcus et al., 2007; Das et al., 2011), but they do not offer a module for participants to complete their own assessments.

**Figure 1:**
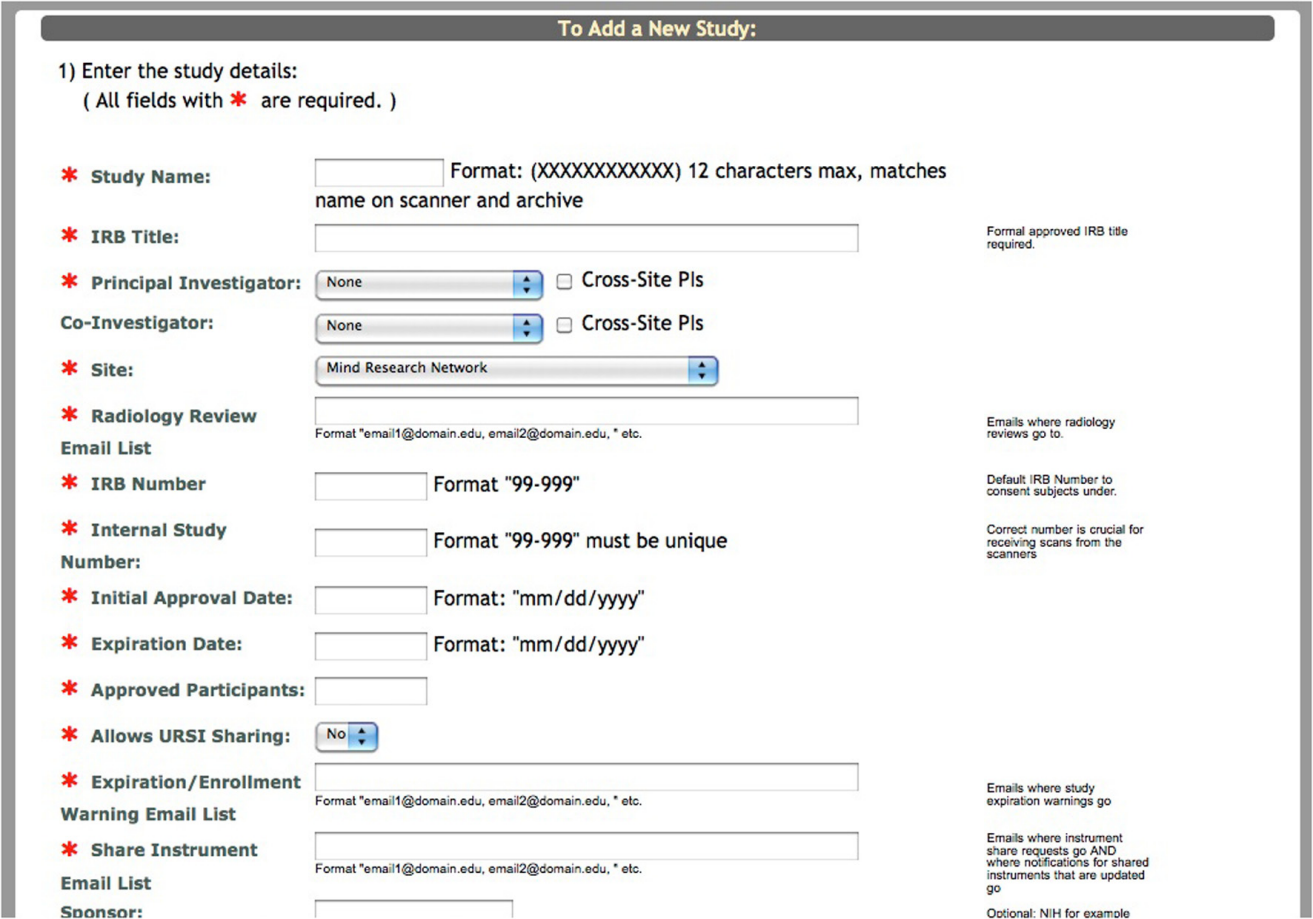
**Add a new study**.

For the purpose of conveying where the Self Assessment tool fits into the study schema of COINS, we will briefly explain the process of adding a new study and enrolling participants in the system. Creating a study involves entering basic information about the study into a form (Figure 1). After this form has been submitted, the research staff must create study visits and subject types for the study. It is important to create study visits that reflect the study protocol. The study visits are used to associate assessments and scans with the time point on which the data was collected. Subject types are the different subject groups in the study protocol (e.g., Smoker, Non-Smoker).

**Figure 2:**
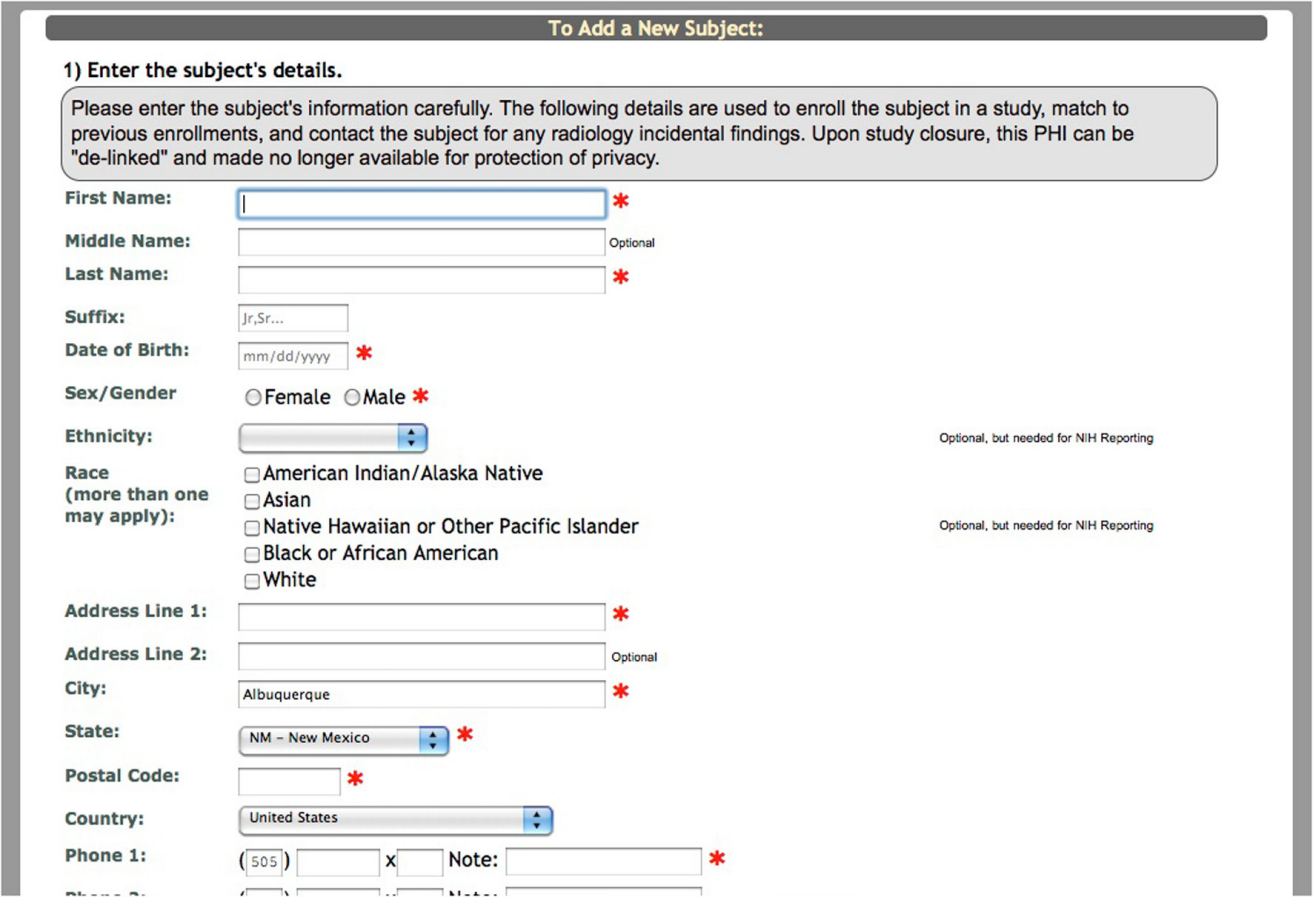
**Add a new subject**.

**Figure 3:**
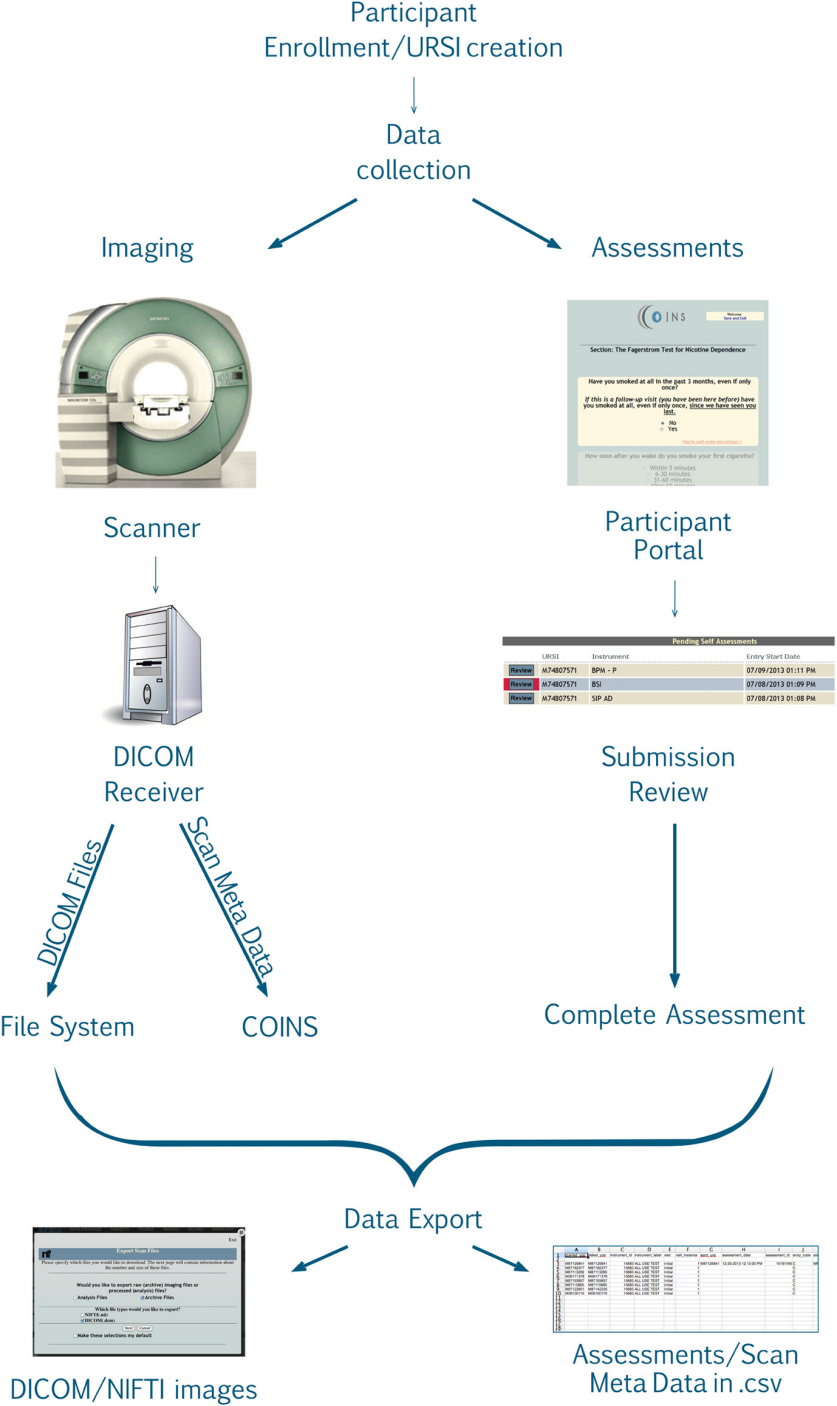
**Data collection flow chart**.

After the study is set up, the research staff can begin enrolling participants into the study. Basic demographic information is entered for each participant during enrollment (Figure 2). At this time a subject type, chosen from the previously created subjects types, is assigned to the participant. Every time a participant is enrolled into a new study, they are assigned a study specific subject ID called an URSI (Unique Research Subject Identifier). In COINS all of the participant data (scans and assessments) are coded with the URSI and are linked together in data collection, data storage and at export (Figure 3).

### Permission levels

The COINS database has been designed with security and restricted/controlled access features. External access to the system is restricted to either a VPN account with the Mind Research Network (MRN) or through a firewall rule for limited IP addresses within a collaborating institution. At the MRN, each user is given a dedicated, password protected COINS account that is only granted after all institutional human protections trainings have been completed. Account renewal is done annually and is dependent on human protections recertification. User access is based on study and role permissions. In order to gain access to a particular study, investigators need to have IRB approval to access that study within the database. The study PI will determine the level of study permissions the investigator is assigned based on their role (e.g., data entry, coordinator, co-investigator, etc.). Certain roles and applications allow an investigator to have access to a study, but not the participant identification details.

COINS is currently in compliance with HIPAA Privacy and Security Rule (Health Insurance Portability and Accountability Act of 1996, 2002) requirements. PHI is encrypted using the mcrypt libraries. The data exists on a virtual machine within the MRN firewall such that access to the machine is limited to only COINS system developers and IT personnel. Permission is granted by the site administrator on a granular level within each study. Raw data is restricted through user permissions both at the filesystem and the web application levels. Raw data, when viewed independent of the associated meta-data, is free of demographic PHI. PHI will be stored encrypted on a PostgresSQL database within the MRN network and protected by its firewall. Participant names and other identifying information will be maintained in this restricted database, available only to authorized members of the research team for the duration of the study. At the time of study closure, the link to participant names and other identifiable data will be unlinked and made inaccessible to the research team.

### Instruments

Clinical data gathered from interviews, questionnaires, and neuropsychological tests are entered into COINS through the Assessment Manager (ASMT) application. The Self Assessment tools are accessible through this application.

One of the first steps to assessment data collection in the COINS database is instrument creation. The term instrument here means the measure (or blank form) through which assessment data is entered (by the research staff or directly by the participants). Instruments can be created in several ways. There is an instrument creation tool in which the general properties of the instrument are entered (instrument label, description, version, etc.), then sections are created manually as well as questions/responses. Another option to create an instrument is the instrument import tool. This process involves the research staff creating a template of the instrument in a.csv file that includes all of the fields that are required during manual instrument creation (instrument properties, sections, questions, etc.) which is then imported into the study by the COINS staff. The final option is to request an existing instrument be shared or copied to the investigator's study.

### Self assessment question types and features

During instrument creation there are several features that can be employed to optimize the participant's experience. Instrument creation in COINS takes into account the need for participant friendly language. For this purpose there are Self Assessment specific instrument, section, and question labels available. These labels can be used in place of stigmatizing language that might influence a participant's responses.

**Figure 4:**
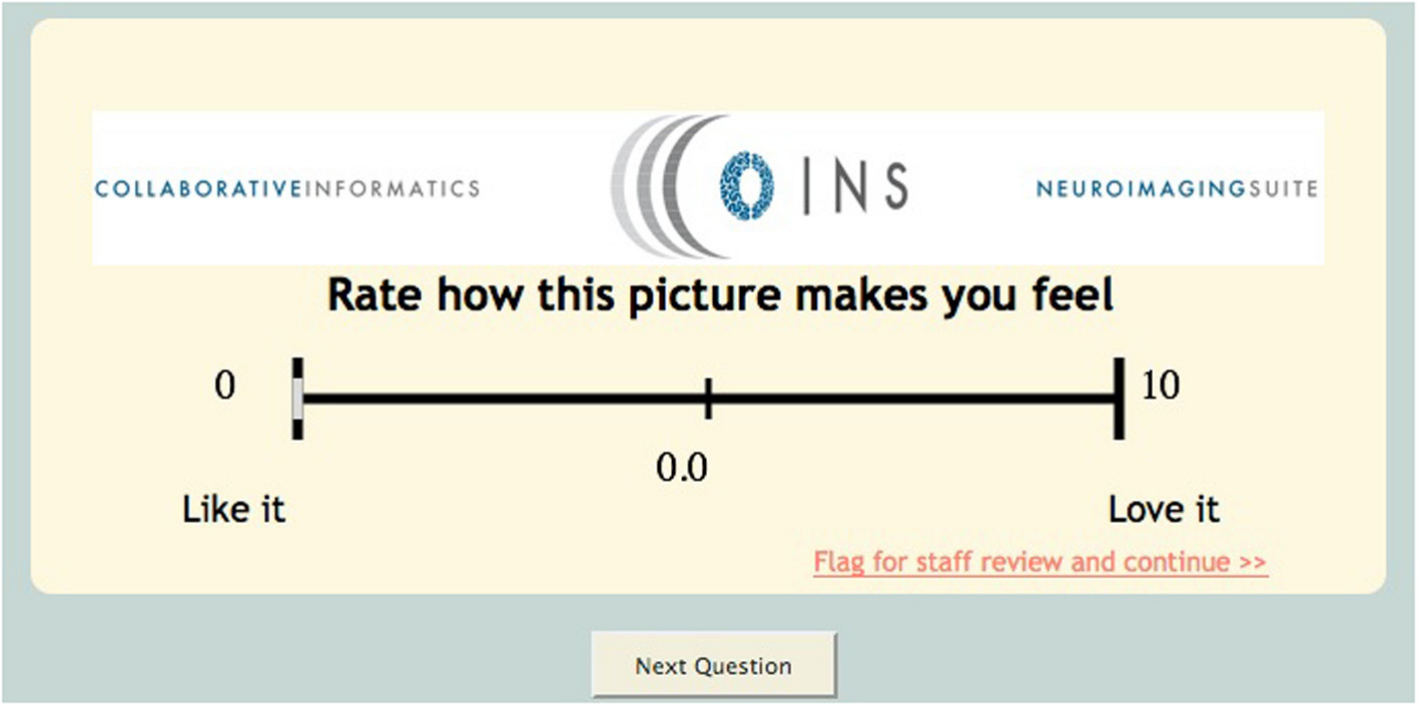
**Visual analog scale**.

This tool also has several different unique question types/features such as media question types. This question type can be used if the investigator would like to capture a participant's response to an image or video. The research staff can upload an image or video and create associated questions (multiple choice, visual analog scale, text response) (Figure 4). An extension of this question type is the continuous visual analog scale (VAS). Continuous VAS questions can be configured to record values at regular intervals while the participant is viewing a video. This can be used to allow participants to rate their emotional response to images/sounds in a video over time.

**Figure 5:**
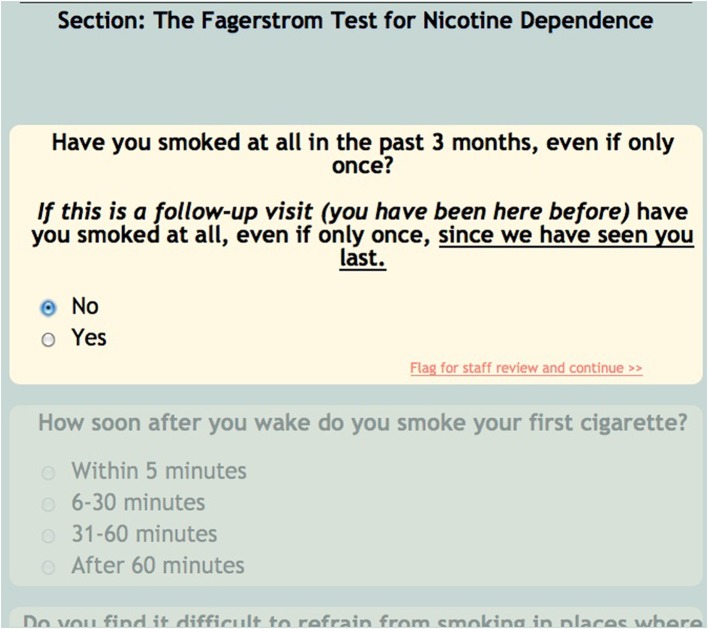
**Conditional skipping**.

**Figure 6:**
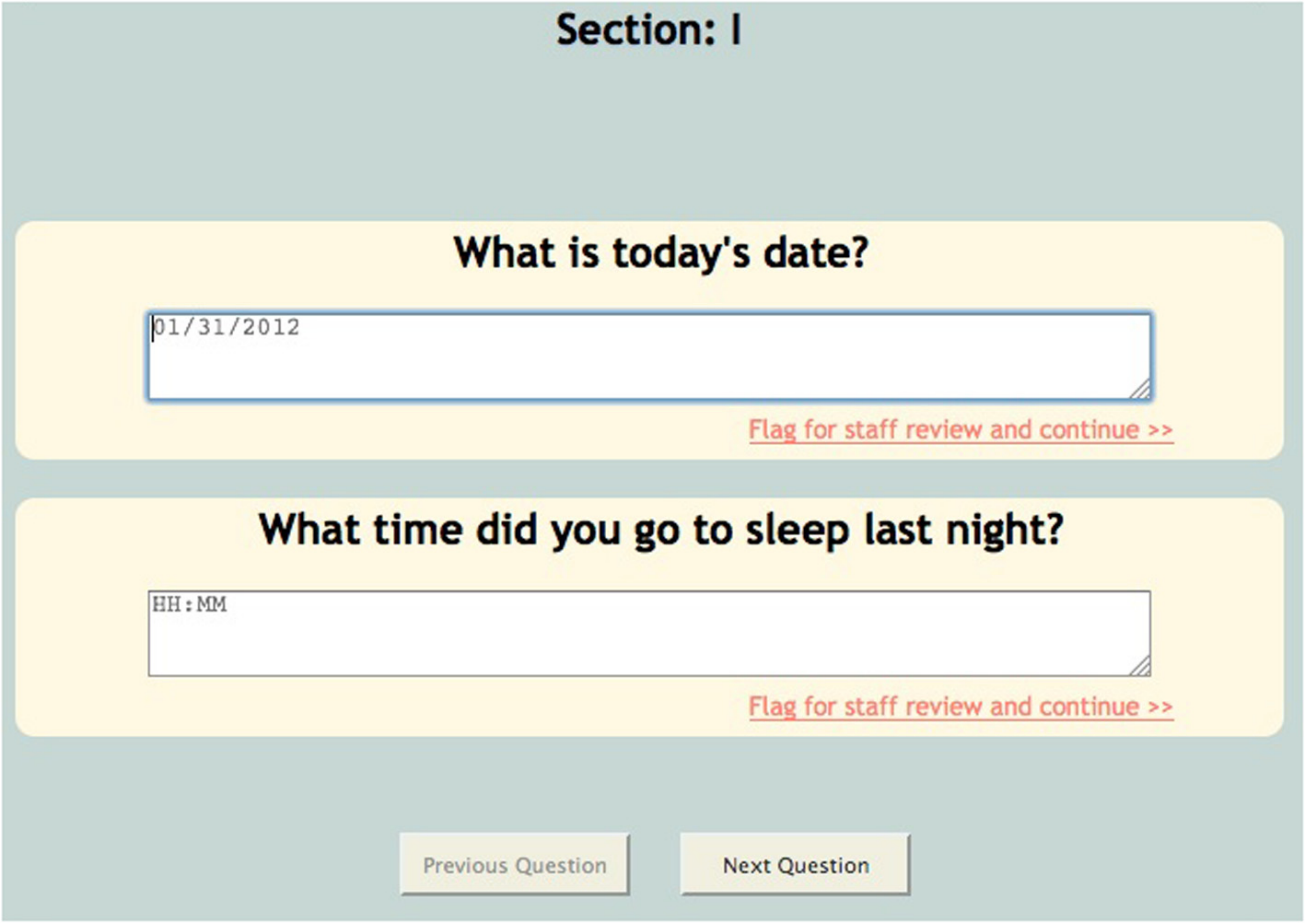
**Text enforcement**.

For the sake of efficiency and accuracy, COINS provides conditional looping, conditional skipping and auto-populated responses. Conditional looping and skipping (also sometimes referred to as “branching logic”) allow the participant to move through a questionnaire without having to answer irrelevant questions (Figure 5). For example, a participant could skip out of answering cigarette smoking related questions if they do not smoke. Auto-populate questions can be used if more than one instrument asks the same question, for example, age. If the participant enters their age in the “auto-populate from” question, the “auto-populate to” question will display that response when the participant reaches that question. This reduces time of entry as well as frustration on the participant's part. The research staff can also choose to make the questions required, this option will not allow the participant to navigate away from a page until all questions are answered. This ensures that the assessment is fully completed. For questions that capture text responses, there are text enforcement options. These can be employed to be sure the correct type (date, phone number, number, time (HH:MM), etc.) of text response is entered (Figure 6).

**Figure 7:**
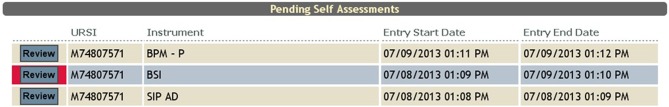
**Critical flagging in review queue**.

**Figure 8:**
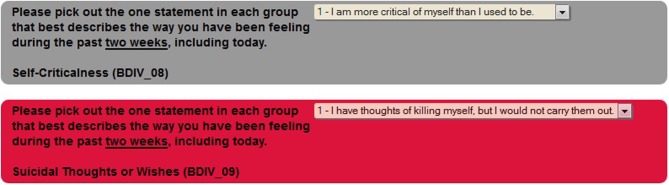
**Critical flagged question**.

Often times neuroimaging research involves asking participants sensitive questions. The responses to these questions could lead to necessary intervention by the research staff (i.e., discussing suicidality). In order to alert the staff to such questions, there is a critical flagging feature that allows any response to be considered a critical flagged response. If such a response is selected by the participant, the research staff will see the assessment in red in their review queue (Figure 7), as well as the critical response question (Figure 8).

### Self assessment preview

To ensure that the instrument presented to the participant functions (i.e., conditional skips, loops, specify options, etc.) as expected there is a tool called SA (Self Assessment) Preview. This tool allows research staff to view the instrument as a participant. Use of this tool is highly recommended for any instrument that will be viewed by participants. The research staff can view the instrument in SA Preview by the click of a button (available on each question), which launches a modal pop up. The instrument is displayed just as it would be to the participant.

### Self assessment queues

In order for participants to fill out the assessments in the Participant Portal the research staff has to populate the “Participant Queue.” Within ASMT there is a “Manage Subject Queues” tool. The user selects the participant's URSI, the study visit (e.g., Baseline, Visit 1, Visit 2) for which they want data entered, the queue type and then creates a login for the participant (it is recommended that these logins not contain any participant identifiers). Queue types determine how the assessment is handled in the Participant Portal. A one-time queue is used for assessments that are only to be collected once per visit. An on-going queue is used if the assessment data is collected throughout the study (e.g., calendar data). Once the data collection is over only the research staff can complete an assessment in the on-going queue. A recurring queue is used if an assessment needs to be collected more than once per visit. Each time the participant opens an assessment in this queue a new assessment is begun and an new instance is created in the database. As with the on-going queue only the research staff can complete an assessment in this queue type.

**Figure 9:**
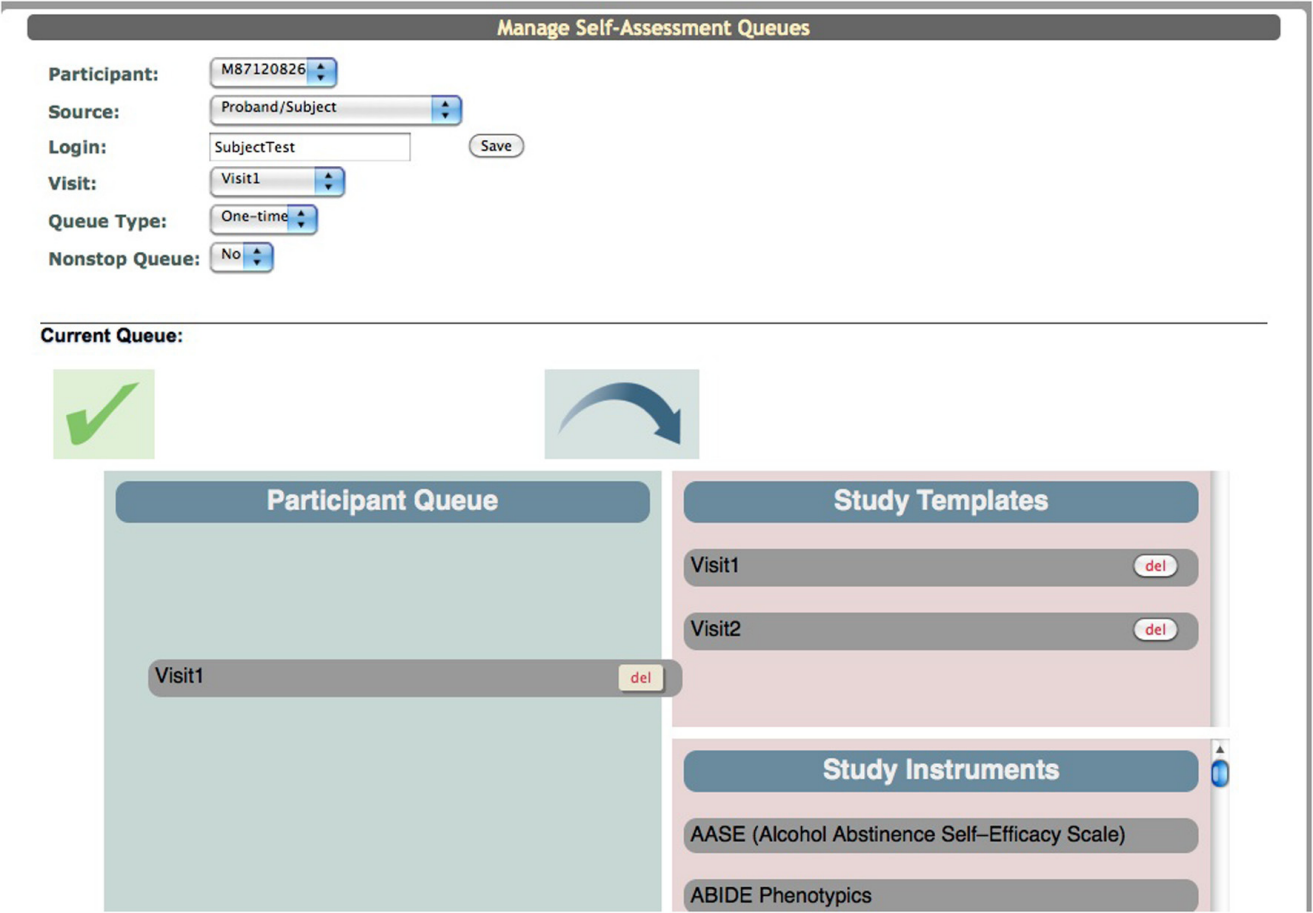
**Participant Queue**.

Creating a queue is a drag and drop system. The interface displays a box for the “Participant Queue,” “Study Templates,” and “Study Instruments” (Figure 9). The “Study Instruments” box includes all of the instruments that have been created for the study. To populate the queue the research staff has to click each desired instrument and drag it from the “Study Instruments” box to the “Participant Queue” box and release. When all instruments have been queued, they save the list and can provide the participant with the website (coins.mrn.org/p2) and login.

### Templates

A template schema was created for ease of use. The user can drag and drop all of the instruments into the “Participant Queue,” click a button and a pop up appears that asks for a template name. The template then appears in the “Study Templates” box. When the next participant is ready to be queued for assessments, the research staff can drag the template previously created over to the “Participant Queue” and the instruments will appear in the same order in which they were saved. This reduces the amount time that it takes for the user to set up the queue as well as accounts for any potential error (forgetting an instrument, adding two of the same instrument, etc.).

### Participant portal

A participant can begin filling out the queued assessments as soon as they login into the Participant Portal. The Participant Portal can be accessed anywhere with an internet connection. The portal has been designed to have an easy to use interface for all ranges of participant types, from those that are computer savvy to those that have had little exposure to computers.

As the participant is completing the assessments, they are made aware of their progress. At the bottom of the screen there is a note to the user indicating how many assessments they have to complete. Also at the end of each assessment there is a brief message that they have completed the assessment and indicates how many assessments are left in their queue. If the participant needs a break, they can click “Save and Exit” and when they log back in they will be brought the last unanswered question.

As the participants complete the assessments the research staff receive emails indicating that an assessment is complete and waiting to be reviewed in the review queue. These emails also contain a link to the review queue so that the researcher can easily access it. In order to receive the notification emails, the research staff enter the desired email addresses in a list during study set up. This list can be edited throughout the duration of the study so that only those who need to, receive the assessment emails.

### Customized CSS in self assessment

**Figure 10:**
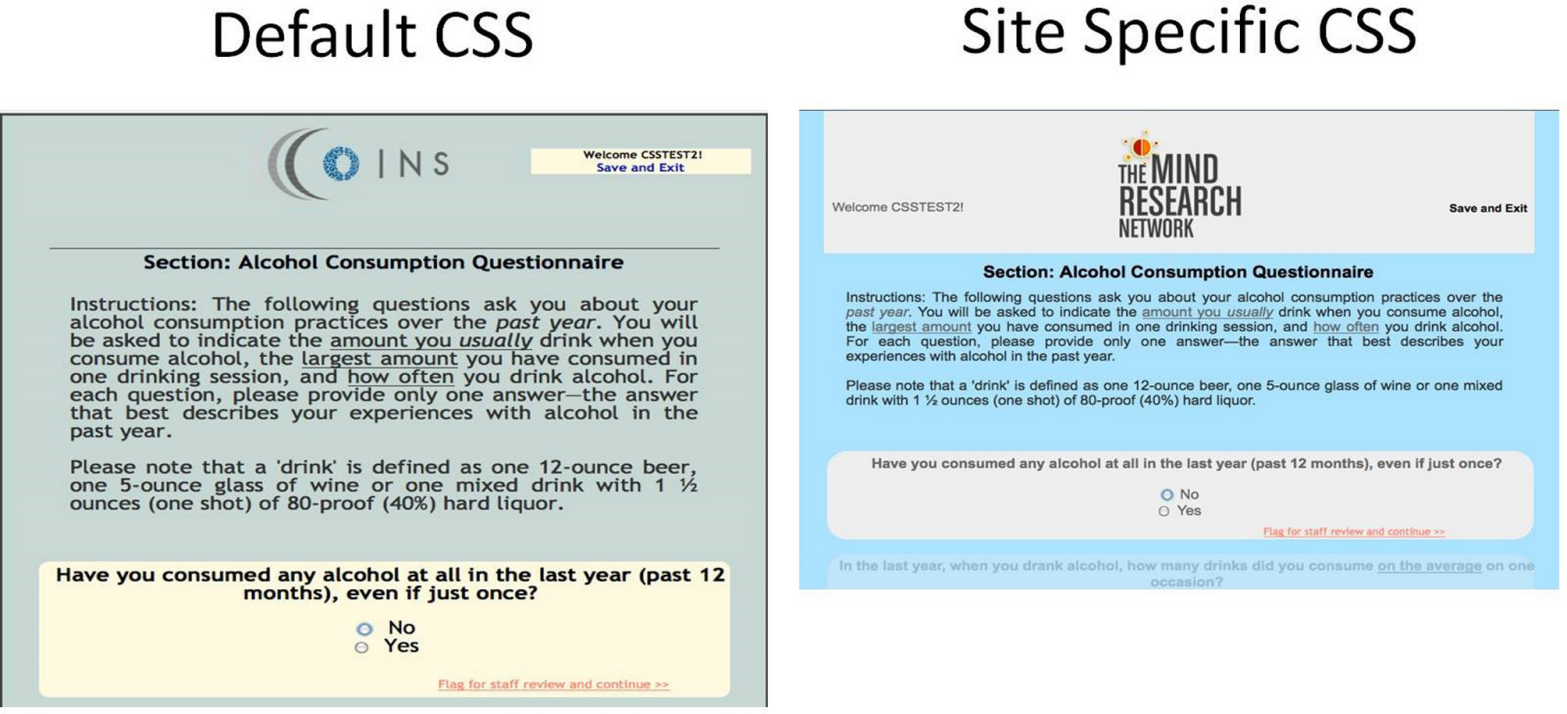
**Participant Portal**.

At the site level, users can customize the Participant Portal with a CSS upload tool. With a basic understanding of CSS, users create a CSS file to change the layout, background, color, and fonts of any generic element or specify a class or id to change more specific elements. They can also upload a logo or graphic from their institution to be displayed at the top of every page in Self Assessment. These tools provide the participant with a feeling of continuity as the Participant Portal will have the same look and feel of the other websites that they are using during their study participation (Figure 10).

### Self assessment review queue

The review queue contains all of the self assessments that have been completed by participants. The research staff member reviewing the assessments has the option to complete the assessment, deny the assessment or save the assessment to review later. If there are no issues with the assessment the study staff can click “Complete” to send the assessment to the database as a finished, complete record (no further entry is needed). If the assessment is incomplete or there is a response that needs clarification, the assessment can be denied. When an assessment is denied it is sent back to the participant's queue for completion/updating. If the user cannot complete the review, they can save it and escape the assessment in order to keep it in their review queue to be reviewed and completed/denied at another time.

**Figure 11:**
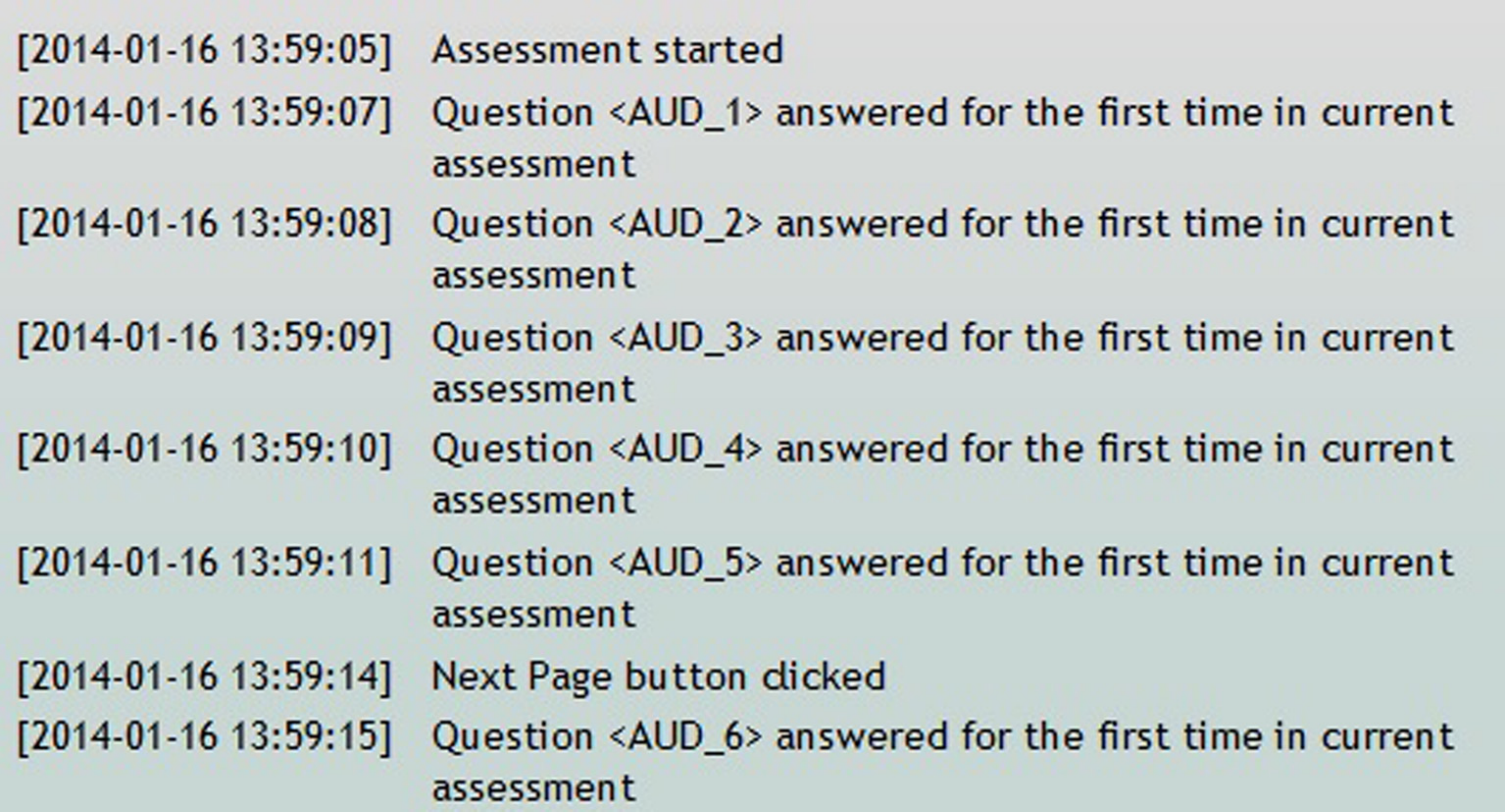
**Self assessment time log**.

The Self Assessment time log is a tool to determine how long participants spend on individual questions or pages while completing an assessment in the Participant Portal. There is a list of all of the self assessments for the study. Included on that list is a column labeled, “time spent,” which displays the time, in minutes, that it took the participant to complete that specific assessment. The user can also view a further breakdown of the time log that displays timing information on every event completed in the assessment (e.g., assessment resumed, question answered, next page button clicked, assessment complete, etc.) (Figure 11).

### Exporting data

Data collected via SA is easily retrieved and exported from Query Builder and/or a study portal. Query Builder is the most versatile data export tool currently offered in the COINS tool suite. This tool supports secure, *ad hoc* querying of single and cross-site studies for assessments, scans and demographics. It also offers the ability to search assessment data and scan data in the same query.

A study portal is a centralized collaboration tool for monitoring enrollment progress, quality assurance, document exchange, etc. Progress reports within the portals provide a complete workflow overview of a study to identify missing data at one glance. Internal and external collaborators can use the portals to access all assessment data associated with their studies. Assessment data can be exported by subject type/instrument/study visit. There are also reports that display graphical views of question scores, demographic statistics and outliers in the data.

## Results

Since the release of the Self Assessment tool in 2011, there have been 35,448 assessments collected across 6 sites (Table 1). Several studies/programs have been instrumental in the continual development of this tool. The enhanced Nathan Kline Institute - Rockland Sample (NKI-RS) is an ongoing project aimed at collecting 1,000 or more participants to provide a lifespan sample (ages 6–85 years old) of phenotypic, neuroimaging and genetics data (Nooner et al., 2012). The initial development of Self Assessment was guided by the expected types of assessments collected by the NKI-RS project. Currently, NKI-RS almost exclusively collects assessment data via Self Assessment, sometimes collecting over 15 assessments at one visit.

**Table 1:** **Assessments per site**.

**Sites**	**Number of assessments**
The Mind Research Network	20,613
Nathan Kline Institute	12,136
NM works—ICARE	2166
University of North Carolina—Wilmington	286
University of Colorado Boulder	238

**Figure 12:**
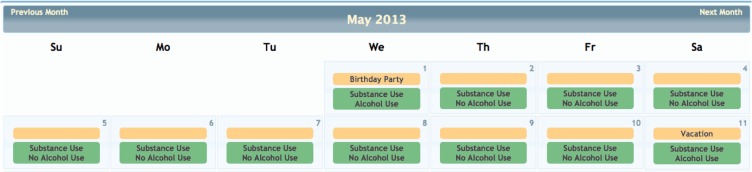
**ICARE calendar**.

**Figure 13:**
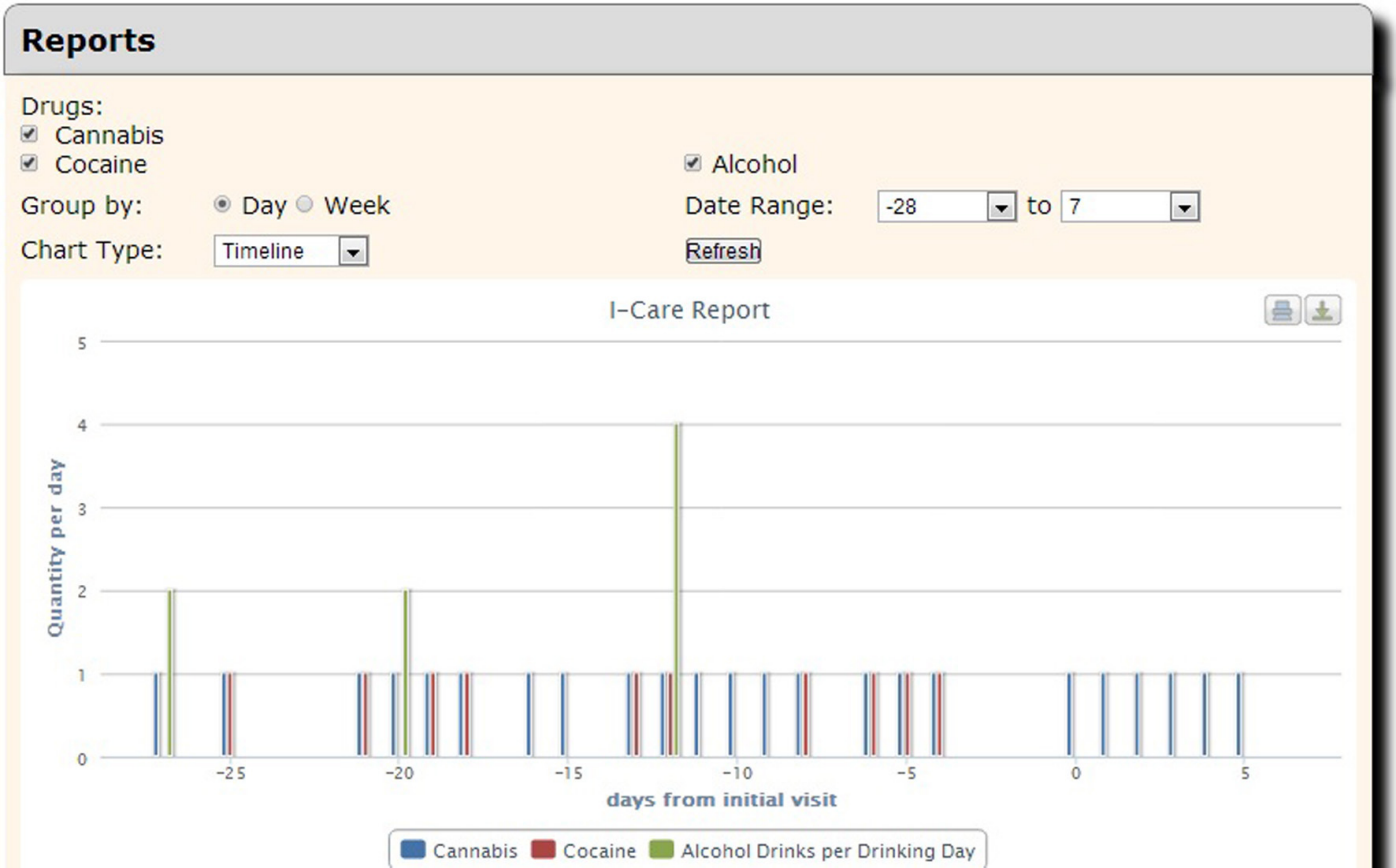
**Calendar report tool**.

Although COINS allows cross modality data collection, not every group using COINS collects imaging data. The New Mexico Works Intensive Case Management, Recovery and Employment (ICARE) program is a pilot program designed to address substance use barriers to employment in Temporary Assistance for Needy Families (TANF) recipients (NM Human Services Department, 2012). The substance abuse data is collected via an SA calendar tool that has been tailored to the Timeline Followback assessment (Sobell and Sobell, 1992). This particular calendar tool is designed to collect life events as well as substance use information for a complete emulation of a paper and pencil Timeline Followback assessment. Data entered by the day into the virtual calendar (Figure 12) can be duplicated, edited and deleted. Multiple days with same information can be entered all at once via simple key commands. This tool is capable of continuous entry when queued in an On-going queue type. The Followback Calendar includes an administrator tool that allows staff to edit previously entered data in the event of an entry error or incorrect reporting. The information entered into the calendar through Self Assessment can then be viewed and exported in very detailed and easy-to-use reports. Substance use information from the calendar can be graphically viewed in several different charts types (Figure 13) via the “Calendar Report Tool.” Each unique substance reported on the calendar can be shown or hidden with toggle icons and can be viewed as a simple, clean bar graph or as a cumulation graph, showing length of use and abstinence periods. All life events and substances used are also plainly listed by date for easy review. In addition to the ability to effortlessly visualize the substance use data, it can also be exported for analysis, allowing for the day range, days per interval customization. This project serves participants that have little or no knowledge of computers and thus far there have not been any barriers during their use of the tool. There have been 2166 self assessments collected for this project thus far.

## Discussion

COINS is under constant development in order to satisfy the need for a database that provides tools for all aspects of a research study. Although we offer a robust tool suite there are several areas in which we can improve and provide more features.

### Auto-queues

We plan to continue to reduce ways in which the research staff have to manually enter information. We are currently developing an auto assessment-queuing process. This tool will enable research staff to set up conditions for automated assessment queues (currently done manually by research staff) based on subject types and/or responses to specific questions. This will reduce errors (queuing incorrect assessments, queuing for incorrect visits, etc.) and the amount of time spent by research staff.

### Offline data storage

Data collection is often conducted in the field, where wireless internet can be unpredictable or non-existent. COINS currently offers a Windows-XP-Tablet-based direct entry application that uses a web service to sync assessment data to the database when a data connection is available (Turner et al., 2011). This application is primarily used by research studies that need to collect data in an environment where no data connection is available (e.g., correctional institutions or rural populations). This tool has proven extremely useful for this purpose and requires little maintenance after a study has been set up. Unfortunately, the application was designed for use exclusively on touch-based Windows XP devices. These devices will no longer be supported by Microsoft in the spring of 2014, and newer tablet technology from Apple, Microsoft and Google warrants a new offline-capable system.

To this end, we plan to leverage HTML5 web standards such as the Local Storage API (Hickson, 2013), and Cache Manifest (HTML5, 2012). This will allow any device with a browser to cache instruments for use in an offline environment, and then store data entered into those instruments on the device until a data connection can be established. Once a data connection is established, data will be synced to the COINS servers, where it can be inspected, approved and imported.

## Conclusion

There are several options available to researchers for assessment data collection, but very few that offer a full neuroimaging tool suite as well as participant entered assessments. The COINS Self Assessment tool is optimal for participant data collection due to its ease of use (for participants and research staff), integration capability with other neuroimaging data, security features for protecting sensitive/identifying participant information. The COINS team will continue to improve the usability of current tools as well as aim to provide new features and tools that will allow COINS stand out as a superior alternative to collecting study data with several different databases/systems.

### Conflict of interest statement

The authors declare that the research was conducted in the absence of any commercial or financial relationships that could be construed as a potential conflict of interest.

## Footnotes

^1^SurveyMonkey Inc. Palo Alto, CA. Available online at: www.surveymonkey.com

^2^Qualtrics and all other Qualtrics product or service names are registered trademarks or trademarks of Qualtrics. Provo, UT. Available online at: http://www.qualtrics.com
